# Dose–Response Evaluation of Sugammadex for Reversal of Deep Rocuronium-Induced Neuromuscular Block in Cats

**DOI:** 10.3390/vetsci12121135

**Published:** 2025-11-29

**Authors:** Natália Mesquita Cesnik, Karina D’Angelo Campos, Jéssica Sperandio Cavaco, Carolina Soares Navarro, André Gustavo Alves Holanda, Julia Maria Matera, Aline Magalhães Ambrósio

**Affiliations:** Department of Surgery, School of Veterinary Medicine and Animal Science, University of São Paulo, São Paulo 05508-900, SP, Brazil; ncesnik@usp.br (N.M.C.); karinadc9@gmail.com (K.D.C.); jessi.cavaco@gmail.com (J.S.C.); carolina.navarro@usp.br (C.S.N.); gustavoholanda.50@gmail.com (A.G.A.H.); materajm@usp.br (J.M.M.)

**Keywords:** feline anesthesia, neuromuscular monitoring, sugammadex, dose–response, selective reversal agent, rocuronium, acceleromyography, train-of-four

## Abstract

**Simple Summary:**

Sugammadex is a drug designed to rapidly reverse the effects of rocuronium, a neuromuscular blocking agent commonly used to produce muscle relaxation during anesthesia. This study tested different doses of sugammadex (2, 4, and 8 mg kg^−1^) in anesthetized cats to identify an effective and safe dose for clinical use. Higher doses resulted in faster and more consistent recovery of muscle function, although greater interindividual variability in the reversal time was observed with the 4 mg kg^−1^ dose. No adverse effects were observed. These findings provide practical guidance for veterinarians, showing that sugammadex can enhance anesthetic safety and ensure a quicker, smoother recovery of muscle activity in feline patients after surgery.

**Abstract:**

**Background:** Sugammadex is a selective γ-cyclodextrin compound that encapsulates steroidal neuromuscular blocking agents such as rocuronium, allowing rapid and predictable recovery from neuromuscular block (NMB). However, dose–response information in feline patients remains limited. **Methods:** In this prospective, randomized, and blinded experimental study, three intravenous doses of sugammadex (2, 4, and 8 mg kg^−1^) were compared for the reversal of profound rocuronium-induced NMB (0.6 mg kg^−1^) in thirty adult ASA I cats anesthetized with sevoflurane. Neuromuscular function was continuously assessed using acceleromyography (train-of-four stimulation). The onset and recovery times for T_1_/T_0_ ratios of 25–90%, T_4_/T_1_ ratios, and recovery index were measured, along with cardiovascular and respiratory parameters. **Results:** Sugammadex shortened the recovery time in a dose-dependent manner. The mean time to achieve T_1_/T_0_ = 90% was 519 s (2 mg kg^−1^), 300 s (4 mg kg^−1^), or 256.8 s (8 mg kg^−1^). The 43-s difference between the two higher doses was not statistically significant (*p* = 0.317) and, therefore, not clinically relevant. Greater interindividual variability in the reversal time was observed at a dose of 4 mg kg^−1^. One cat in this group experienced transient recurarization, and no adverse cardiovascular effects were detected. **Conclusions:** Both 4 and 8 mg kg^−1^ of sugammadex produced rapid and complete reversal of profound rocuronium-induced NMB in sevoflurane-anesthetized cats without hemodynamic compromise. These results apply to healthy ASA I cats, and further studies are warranted in animals with systemic disease.

## 1. Introduction

Neuromuscular blocking agents (NMBAs) are frequently used in veterinary anesthesia to facilitate intubation, optimize surgical conditions, and improve mechanical ventilation control. Among the non-depolarizing agents, rocuronium has become widely applied due to its intermediate duration of action and predictable pharmacological profile. Nevertheless, spontaneous recovery from rocuronium-induced neuromuscular blockade (NMB) can be slow and variable, predisposing patients to residual paralysis and postoperative complications. In human medicine, incomplete recovery of neuromuscular function has been associated with hypoxemia, aspiration pneumonia, and delayed extubation, emphasizing the importance of precise monitoring and effective reversal strategies [1]. In veterinary species, however, data on these complications remain scarce.

The traditional reversal of NMB relies on acetylcholinesterase inhibitors, such as neostigmine, which are effective only for mild to moderate blockades and can cause undesirable cholinergic effects [2]. The development of sugammadex, a modified γ-cyclodextrin, represented a significant advancement in anesthesia pharmacology. This molecule encapsulates and inactivates steroidal NMBAs—including rocuronium and vecuronium—forming stable water-soluble complexes that are excreted unchanged by the kidneys [3,4,5]. The result is a rapid, reliable, and dose-dependent reversal of even profound blockade, without the adverse effects observed with neostigmine or anticholinergic co-administration [6].

Experimental investigations have demonstrated the efficacy of sugammadex in dogs [7,8], horses [9], and cats [10,11]. However, significant species-specific physiological differences may influence the pharmacodynamic response to neuromuscular blocking agents and their reversal. Compared with dogs and horses, cats exhibit slower hepatic metabolism, differences in acetylcholine receptor density, and greater sensitivity to steroidal NMBAs, which can modify both the onset of and recovery from blockade. Additionally, variations in renal clearance and anesthetic–drug interactions may contribute to distinct reversal kinetics in feline species, highlighting the need for targeted dose evaluation [7,8,9,10,11,12]. In humans, a dose of 4 mg kg^−1^ is recommended for the rapid reversal of deep blockade [12]; however, interspecies differences in neuromuscular physiology and anesthetic interactions limit direct extrapolation to veterinary patients. Furthermore, the influence of inhalational anesthetics such as sevoflurane on the pharmacodynamics of both rocuronium and sugammadex in cats has not been fully elucidated.

Therefore, the present study aimed to determine the dose–response relationship of sugammadex for reversing profound rocuronium-induced NMB in healthy cats anesthetized with sevoflurane. By comparing three dosing regimens, 2, 4, and 8 mg/kg^−1^, we aimed to establish evidence-based guidelines for the safe and effective clinical application of sugammadex in feline anesthesia. We hypothesized that 4 mg kg^−1^ would provide a plateau in reversal efficacy, achieving rapid and consistent recovery, without significant benefit from higher doses.

## 2. Materials and Methods

### 2.1. Animals

This prospective, randomized, and blinded study was approved by the Institutional Animal Care and Use Committee (CEUA no. 1743091023) of the School of Veterinary Medicine and Animal Science, University of São Paulo, and conducted in accordance with ARRIVE guidelines. Thirty healthy, mixed-breed cats (twenty-one males and nine females), aged 17 ± 10 months and weighing 3.8 ± 0.67 kg, were enrolled after clinical and laboratory examination and classified as American Society of Anesthesiologists (ASA) physical status I. Food was withheld for 8 h and water for 2 h before anesthesia. All animals underwent elective ovariohysterectomy or orchiectomy. All the cats were client-owned animals admitted for elective procedures at the Veterinary Teaching Hospital, and their welfare was ensured throughout hospitalization and recovery, in accordance with institutional and national animal care standards.

The sample size was calculated using an online power analysis tool (http://powerandsamplesize.com/Calculators/Compare-k-Means/1-Way-ANOVA-Pairwise, accessed on 22 November 2025), assuming 95% power and α = 0.05 based on pilot data for the T_1_ 90% recovery variable. Random allocation was performed by means of a sealed-envelope draw, assigning animals equally (*n* = 10 per group) to receive 2, 4, or 8 mg kg^−1^ of sugammadex. No blocking or stratification by sex or body weight was applied, as animals were evenly distributed across the groups. The investigator responsible for data collection was blinded to group assignment.

### 2.2. Anesthesia and Monitoring

All cats were premedicated intramuscularly 30 min before induction with a combination of buprenorphine (0.02 mg kg^−1^; Beniv^®^, Ourofino, Cravinhos, SP, Brazil) and ketamine (1 mg kg^−1^; Cetamin^®^, Syntec, Santana do Parnaíba, SP, Brazil). Anesthesia was induced with intravenous propofol (5–8 mg kg^−1^; Provive^®^, União Química, São Paulo, SP, Brazil), administered slowly until muscle relaxation and loss of the palpebral and laryngotracheal reflexes were achieved. During the anesthetic procedure, lactated Ringer’s solution was infused intravenously at a constant rate of 3 mL kg^−1^ h^−1^ to maintain adequate hydration.

Anesthetic maintenance was performed with sevoflurane (Sevocris^®^, Cristália, São Paulo, SP, Brazil), with end-tidal concentrations between 2.0 and 3.0% in 60% inspired O_2_, using a circle system and pressure-controlled ventilation set to deliver 8–10 mL/kg tidal volume; the respiratory rate was adjusted to maintain normocapnia (ETCO_2_ 30–40 mmHg). The vaporizer setting was adjusted according to eye position, muscle tone, and cardiovascular parameters, and the end-tidal sevoflurane concentration (P’E_SEVO) remained constant throughout the neuromuscular evaluation and data collection. Individual MAC values were not predetermined for each animal before the study.

Physiological variables were continuously monitored using a multiparameter device (Triton BSM-6000^®^, Nihon Kohden, Tokyo, Japan). The variables included heart rate (HR); systolic, diastolic, and mean arterial pressure (SAP, DAP, and MAP, respectively); respiratory rate (RR); peripheral oxygen saturation (SpO_2_); end-tidal carbon dioxide (P’E_CO_2_); fractional inspired oxygen (FiO_2_); and esophageal temperature (T). All parameters were recorded at 5-min intervals.

For invasive blood pressure measurements, a 24-gauge catheter (Safelet^®^, Nipro Medical, São Paulo, SP, Brazil) was inserted into the dorsal metatarsal artery. When the mean arterial pressure dropped to ≤65 mmHg, the sevoflurane concentration was reduced, and a bolus of lactated Ringer’s solution (10 mL kg^−1^ over 10 min) was administered. If the response was inadequate, ephedrine (0.1 mg kg^−1^ IV) was administered as a rescue therapy.

### 2.3. Neuromuscular Blockade Assessment

Neuromuscular transmission was evaluated using acceleromyography with an NMT module (AF-101P, Triton BSM-6000, Nihon Kohden, Tokyo, Japan). Each cat was positioned in dorsal recumbency. The negative electrode was placed over the ulnar nerve at the medial aspect of the elbow, while the positive electrode was positioned approximately 2 cm proximally. The acceleration transducer was attached to the palmar surface of the distal phalanges of the same limb, allowing free movement of the paw (Supplementary Video S1). The skin temperature of the monitored limb was maintained at a minimum of 35 °C using a forced-air warming system (Warm Air, Gentherm, Novi, MI, USA) to minimize variability in acceleromyography responses and prevent hypothermia.

Following instrumentation, train-of-four (TOF) stimulation was initiated with the following parameters: 0.2 ms pulse duration, 2 Hz frequency, and a 15-s cycle. Baseline measurements were obtained after a stabilization period of approximately 15 min. Calibration of the device was performed automatically before the administration of any neuromuscular blocking agent. TOF stimulation continued at regular 15-s intervals throughout the experiment, and all responses were recorded manually.

The following variables were evaluated:

- lagROC—the time from the start of rocuronium injection to the initial decrease in twitch amplitude;
- onsetROC—the time from rocuronium administration to complete suppression of the first twitch;
- lagSUG—the time from the start of sugammadex injection to the reappearance of the first twitch;
- T_1_/T_0_ recovery to 25%, 50%, 75%, and 90%;
- Recovery index—the interval between 25% and 75% T_1_ recovery;
- TOF ratio = 0.9—the time required for the TOF ratio to reach 0.9 after sugammadex administration.

### 2.4. Administration of Rocuronium and Sugammadex

After calibration and stabilization of the neuromuscular response, all cats received an intravenous bolus of rocuronium (0.6 mg/kg^−1^; ROCuron^®^, Cristália, São Paulo, SP, Brazil) administered over 5 s. Profound neuromuscular blockade was confirmed when the TOF count reached zero and the post-tetanic count (PTC) was ≤2. Five minutes after confirmation of complete blockade, sugammadex (Bridion^®^, MSD, São Paulo, SP, Brazil) was injected as a single intravenous bolus via a cephalic vein catheter over 5 s at the assigned dose of 2 mg kg^−1^, 4 mg kg^−1^, or 8 mg kg^−1^, according to group allocation.

Hemodynamic and neuromuscular parameters were continuously recorded for 30 min following administration of the reversal agent to detect any evidence of residual paralysis or recurarization. Upon completion of anesthesia, the cats were monitored for four hours during recovery to assess respiratory effort, reflexes, muscle tone, and potential adverse reactions.

### 2.5. Statistical Analysis

All data were analyzed using Jamovi software (version 2.3.28.0). Normality of distributions was evaluated with the Shapiro–Wilk test. When data met the assumption of normality, comparisons among the three groups were performed using one-way analysis of variance (ANOVA), followed by Tukey’s post hoc test. For non-normally distributed variables, the Kruskal–Wallis test was applied, with Durbin–Conover pairwise comparisons when appropriate, as these nonparametric methods provide reliable inferences for small sample sizes and non-Gaussian data distributions.

Homogeneity of variances was tested with either Bartlett’s or Levene’s test, depending on the data distribution. The results are expressed as means ± standard deviations (SDs), and statistical significance was established at *p* < 0.05.

## 3. Results

**Figure 1:**
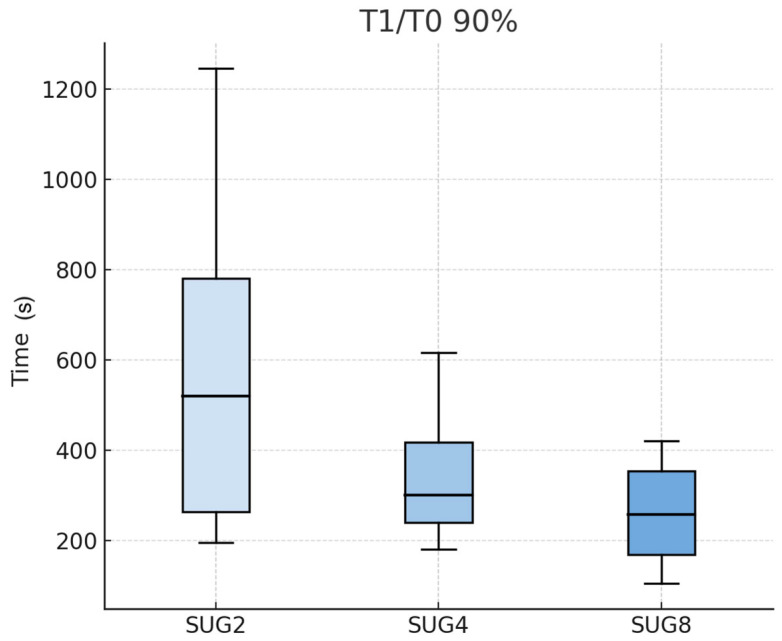
Recovery times for the first twitch to 90% (T_1_/T_0_ = 90%) in seconds after sugammadex administration for sugammadex doses of 2, 4, and 8 mg kg^−1^. Legend: SUG2, 2 mg kg^−1^; SUG4, 4 mg kg^−1^; SUG8, 8 mg kg^−1^ of sugammadex.

Administration of sugammadex resulted in a dose-related shortening of neuromuscular recovery times (T_1_/T_0_ 90%). Although the mean recovery intervals tended to decrease as the administered dose increased, these differences did not reach statistical significance (Figure 1).

The median time from sugammadex injection to restoration of a train-of-four ratio of 0.9 progressively declined across the groups: 52.5 (15–75) seconds for SUG2, 45.0 (15–60) seconds for SUG4, and 30.0 (15–45) seconds for SUG8 (*p* = 0.098). A comparable trend was noted for the recovery of the first twitch amplitude to 90% of baseline (T_1_/T_0_ = 90%), with median (range) values of 519 (195–1245) seconds, 300 (180–615) seconds, and 257 (105–420) seconds for SUG2, SUG4, and SUG8, respectively (*p* = 0.242) (Table 1).

**Figure 2:**
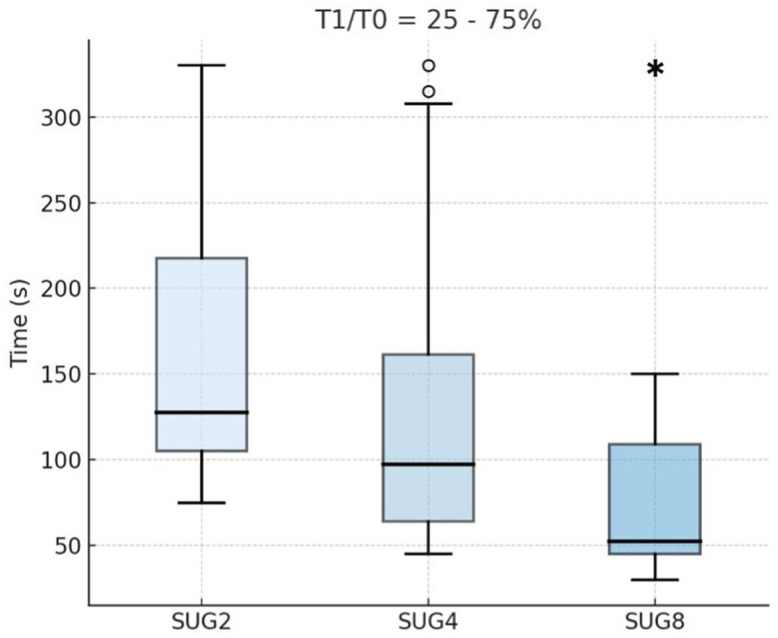
Recovery index (T_1_/T_0_ = 25–75%) values in seconds after sugammadex administration for the groups treated with 2, 4, and 8 mg kg^−1^ of sugammadex. Legend: SUG2, 2 mg kg^−1^; SUG4, 4 mg kg^−1^; SUG8, 8 mg kg^−1^ of sugammadex. Asterisks (*) indicate values significantly different from that for SUG2 (*p* < 0.05). Little circle indicate outlier.

Significant differences were, however, detected at intermediate recovery stages. The SUG8 group recovered more rapidly than the SUG2 group both for T_1_/T_0_ = 75% (*p* = 0.027) and for the recovery index (25–75% interval, *p* = 0.023). These findings demonstrate a faster return of neuromuscular function with higher doses of sugammadex (Table 1, Figure 2).

No significant differences were observed in lag time among the groups (*p* = 0.817). The coefficients of variation (CV%) demonstrated greater variability in the SUG2 group (71.98%) compared with the SUG4 (42.28%) and SUG8 (42.32%) groups.

The time to the return of spontaneous ventilation was similar among the groups: 30.0 (15–75) seconds for SUG2, 30.0 (15–45) seconds for SUG4, and 30.0 (15–30) seconds for SUG8 (*p* = 0.958). Physiological variables showed no statistically significant differences among the groups after sugammadex administration, regardless of the dose (*p* > 0.05) (Table 2). SpO2 levels were consistently above 97% in all cats during the study.

End-tidal sevoflurane concentrations remained stable and comparable among the groups throughout anesthesia (Table 2).

All cats received a crystalloid fluid bolus (10 mL/kg over 10 min), and 17 were administered an ephedrine bolus (0.1–0.2 mg kg^−1^) to maintain MAP > 65 mmHg after induction and before initiation of the experimental protocol, with no intergroup difference (*p* > 0.05).

A single case of transient recurarization occurred in the SUG4 group. In this cat, neuromuscular function initially recovered to a TOF ratio of 0.9 approximately 2.75 min after sugammadex administration, followed by a temporary decline to 0.48 and a subsequent increase to 0.8 at 16.25 min. No respiratory or hemodynamic complications were observed, and the recovery period following extubation was uneventful.

## 4. Discussion

This study provides new evidence regarding the dose–response relationship of sugammadex for reversing profound rocuronium-induced neuromuscular blockade (NMB) in cats anesthetized with sevoflurane. A clear dose-dependent trend was observed: as the dose increased from 2 to 8 mg kg^−1^, recovery became faster and more consistent. Although some differences did not reach statistical significance, this pattern aligns with those from human anesthesia studies that demonstrated more predictable reversal with doses of ≥4 mg kg^−1^ [6].

The mean lag time (16 ± 3.8 s) and onset time (29.5 ± 6.2 s) for rocuronium found here closely resembled those previously described in feline investigations using similar protocols [11]. These data reinforce that rocuronium maintains a relatively short onset and stable pharmacodynamic profile in cats, comparable to observations in dogs and non-human primates [4,6,7,8].

In the present study, the recovery parameters following sugammadex administration—particularly TOF 0.9 (30 ± 3.16 s) and T_1_/T_0_ 90% (257.4 ± 108.6 s)—showed faster return of neuromuscular function in the higher-dose groups (SUG8). This finding aligns with those from earlier canine studies, in which 8 mg kg^−1^ sugammadex reversed rocuronium or vecuronium blockade within one to two minutes [7,13], and with clinical data in humans demonstrating recovery to TOF 0.9 in approximately one minute [14].

Significant group differences were confirmed for intermediate recovery phases (T_1_/T_0_ = 75% and recovery index 25–75%), indicating that cats given 8 mg kg^−1^ regained twitch strength more rapidly than those receiving 2 mg kg^−1^. The 4 mg kg^−1^ dose produced intermediate results, without statistical significance from either extreme, suggesting that this concentration may be clinically sufficient in most cases of profound block reversal under sevoflurane.

Before the present investigation, only one experimental study had explored sugammadex in cats, using 5 mg kg^−1^ and reporting a mean time to T_1_/T_0_ = 90% of 4.7 ± 0.25 min [10]. This outcome is nearly identical to that of the current SUG4 and SUG8 groups (5.0 and 4.3 min, respectively), supporting the reproducibility of feline responses and the appropriateness of using intermediate doses in clinical practice. In contrast, the literature on sugammadex use in humans emphasizes greater interindividual variability, with recovery times ranging from one to more than ten minutes even when identical doses are used [15,16,17].

One cat in the SUG4 group exhibited transient recurarization, with a secondary decline in the TOF ratio after initial recovery. Although isolated, this event underscores the importance of continuous quantitative monitoring after reversal, as spontaneous recurrence of weakness has been reported in both human [18,19,20,21] and veterinary anesthesia [22]. This phenomenon may result from the redistribution of unbound rocuronium from peripheral compartments back into the plasma, an insufficient sugammadex concentration relative to the NMBA load (underdosing), or delayed equilibration of the drug–complex binding process, leading to transient decreases in the TOF ratio despite apparent recovery. These mechanisms underscore the importance of cautious titration and post-reversal vigilance to ensure sustained neuromuscular recovery [22].

No clinically relevant cardiovascular, respiratory or other effects were observed following sugammadex injection, corroborating previous evidence of its hemodynamic stability in other species [23,24]. The maintenance of constant end-tidal sevoflurane concentrations among the groups further confirms that differences in recovery speed are attributable to pharmacodynamic factors rather than anesthetic depth.

Taken together, the first half of the results demonstrates that sugammadex rapidly and safely antagonizes profound rocuronium blockade in cats, with a clear tendency toward faster and more uniform recovery at 4–8 mg kg^−1^. These findings provide essential translational information to guide dose selection and underscore the importance of objective monitoring when neuromuscular blocking agents are used in veterinary anesthesia.

Reversal of residual blockade is a critical component of anesthetic safety. In human medicine, acceleromyography monitoring has been proven to minimize postoperative weakness and enhance recovery quality [18]. Comparable vigilance is necessary in veterinary anesthesia, where residual curarization may remain unnoticed without quantitative assessment. This study reinforces that objective neuromuscular monitoring—beyond subjective evaluation of spontaneous breathing or reflexes—is essential for determining the appropriate timing for extubation in feline patients.

The pharmacological action of sugammadex differs fundamentally from that of anticholinesterase agents such as neostigmine, which are limited to shallow or moderate blocks [12]. By encapsulating rocuronium molecules, sugammadex enables rapid and complete reversal even under deep anesthesia [3,4]. Its dose-dependent efficiency observed in this investigation mirrors that in earlier preclinical trials in monkeys [4] and clinical studies in humans [6,18,19], demonstrating that the drug’s mechanism is highly conserved across species.

The present findings also align with those from canine studies [7,8] and equine trials [9], which showed predictable recovery patterns following similar dosing schemes. The comparable pharmacodynamic response among different veterinary species supports the hypothesis that interspecies differences are primarily quantitative, rather than qualitative, and are related mainly to the distribution volume and renal clearance [5]. In cats, as previously demonstrated [10,11], the duration of action of rocuronium under inhalant anesthesia can be prolonged by the effects of sevoflurane on neuromuscular transmission, highlighting the importance of adequate reversal.

Sevoflurane itself produces cardiovascular and respiratory depression [23,24], which can mask or exaggerate the signs of residual paralysis if neuromuscular recovery is not objectively confirmed. Hypotension occurred in 56% of the cats, which is consistent with sevoflurane’s cardiovascular depression effect [24]. Additionally, pre-anesthetic fasting, insensible fluid losses, and dehydration may have contributed to a volume deficit compromising hemodynamic stability and favoring the observed hypotensive episodes [25]. Importantly, no temporal association was noted between hypotension and sugammadex administration.

A relevant observation in this study was the discrepancy between T_1_/T_0_ = 90% and TOF ratio = 0.9, with longer recovery times for T_1_ in all groups. Sakai et al. [26] previously reported this pattern in cats, with discrepancies of up to 7 min between these parameters. In the present study, the mean variation was 7.9 min with SUG2, 4.7 min with SUG4, and 3.8 min with SUG8. This contrasts with findings in dogs and humans, wherein these parameters recover proportionally [1,27]. This difference may reflect species-specific characteristics of feline neuromuscular junctions, such as a lower safety margin for neuromuscular transmission and slower postjunctional receptor reactivation, resulting in delayed recovery of the first twitch amplitude (T_1_) compared to the TOF ratio. Additionally, technical aspects of acceleromyography, including muscle fiber composition, limb geometry, and motion artifact sensitivity, may contribute to these discrepancies [27]. Such findings reinforce the importance of using multiple monitoring endpoints when assessing recovery from neuromuscular blockade in cats.

The recommended dose for profound block reversal in humans is 4 mg kg^−1^ [2,6]; the present feline data support this as an effective and safe benchmark, with 8 mg kg^−1^ providing no meaningful clinical advantage. Nonetheless, the variability seen at 2 mg kg^−1^ suggests that subtherapeutic dosing may compromise consistency. These findings provide practical guidance for clinical application in cats.

Finally, the absence of cardiovascular or respiratory complications reinforces previous reports of the excellent safety profile of sugammadex in veterinary species [7,8]. The use of balanced crystalloid therapy, as outlined in the 2024 AAHA Fluid Therapy Guidelines [25], likely contributed to hemodynamic stability. Continuous quantitative monitoring, as emphasized by Sakai et al. [26], remains indispensable, since visual or tactile evaluation alone cannot reliably confirm full neuromuscular recovery. Collectively, these observations establish a comprehensive basis for incorporating sugammadex into feline anesthesia, aligning veterinary practices with the precision standards that have long been adopted in human anesthesiology.

This study has several limitations that should be taken into consideration. Although the sample size was sufficient to detect significant differences, larger studies are required to assess the incidence of recurarization and its clinical impact. Therefore, these findings may not be fully generalizable to clinical populations with comorbidities or under different anesthetic conditions. Another limitation was the absence of neuromuscular monitoring during the surgical procedure, which prevented analysis of the potential influence of surgical stimulation on neuromuscular recovery. Additionally, a nerve stimulator was not used for the precise localization of the ulnar nerve, which may have led to minor electrode misplacement and affected the results. Furthermore, plasma concentrations of sugammadex and rocuronium were not measured, precluding pharmacokinetic–pharmacodynamic correlation and potentially contributing to the observed interindividual variability in recovery times. A further limitation is the potential modulatory effect of sevoflurane on neuromuscular transmission, particularly because individualized MAC values were not determined. Finally, the five-minute sampling interval for physiological data represents an additional limitation, as it may have been too infrequent to capture rapid or transient cardiovascular or respiratory changes during the recovery phase.

Future research should further examine the dose–response relationship of sugammadex in cats and assess its applicability under a broader range of clinical conditions. Studies incorporating patients with systemic diseases, diverse anesthetic protocols, and variable intraoperative factors are essential to determine the robustness of these findings. Expanding this line of investigation will contribute to establishing species-specific recommendations for the safe and effective reversal of neuromuscular blockade in feline anesthesia.

## 5. Conclusions

The present study demonstrated that sugammadex effectively reverse profound rocuronium-induced neuromuscular blockade in cats anesthetized with sevoflurane in a dose-dependent manner. Doses of 4 and 8 mg/kg promoted rapid recovery, with T_1_/T_0_ = 90% achieved in 5.00 (3.00–10.25) and 4.28 (1.75–7.00) minutes, respectively. However, one case of transient recurarization was observed at 4 mg/kg, highlighting the need for caution when using intermediate doses. These results apply to healthy cats under sevoflurane anesthesia and may not directly extend to clinical patients with comorbidities or different anesthetic protocols. Continuous quantitative neuromuscular monitoring is strongly recommended to ensure complete and safe recovery from neuromuscular blockade in feline anesthesia.

## Figures and Tables

**Table 1:** Effect of sugammadex on recovery times from rocuronium-induced neuromuscular block (all results in seconds).

	SUG2 (n = 10) Median (Range) (s)	SUG4 (n = 10) Median (Range) (s)	SUG8 (n = 10) Median (Range) (s)	*p*-Value
lagROC	15.0 (15–15)	15.0 (15–30)	15.0 (15–15)	0.126
onsetROC	30.0 (15–45)	30.0 (15–45)	30.0 (30–30)	0.812
lagSUG	22.5 (15–45)	22.5 (15–30)	15.0 (15–30)	0.817
T_1_/T_0_ = 25%	30.0 (15–45)	30.0 (15–45)	30.0 (15–45)	0.732
T_1_/T_0_ = 50%	67.5 (45–135)	60.0 (30–120)	45.0 (15–75)	0.102
T_1_/T_0_ = 75%	165.0 (105–375)	135.0 (75–360)	82.5 * (45–195)	0.027
T_1_/T_0_ = 90%	519.0 (195–1245)	300.0 (180–615)	257.0 (105–420)	0.242
T_4_/T_1_ = 0.9	52.5 (15–75)	45.0 (15–60)	30.0 (15–45)	0.098
T_1_/T_0_ = 25–75%	127.5 (75–330)	97.5 (45–330)	52.5 * (30–150)	0.023

Legend: lagROC—time from start of administration to first decrease in neuromuscular response; onsetROC—time from rocuronium administration to maximum blockade; lagSUG—time from start of sugammadex administration to first detectable twitch; T_1_/T_0_ = 25%, 50%, 75%, 90%—time to recovery of first twitch; recovery index—time from 25% to 75%; T_4_/T_1_ = 0.9—time taken to achieve a train-of-four ratio of 0.9. Values with an asterisk (*) differ significantly (*p* < 0.05) from the corresponding value for the SUG2 group.

**Table 2:** Mean ± SD values of vital signs of thirty cats before and after administration of rocuronium 0.6 mg kg^−1^ (ROC), followed 5 min later by sugammadex 2 mg kg^−1^, 4 mg kg^−1^, or 8 mg kg^−1^ (SUG). No statistically significant differences were observed among the groups.

Times	Baseline	ROC 1	ROC 3	ROC 5	SUG 1	SUG 3	SUG 5	SUG 10	SUG 20	SUG 30
**Heart rate (beats/min)**										
**SUG2**	131 ± 23	141 ± 16	145 ± 16	149 ± 13	150 ± 22	139 ± 15	135 ± 15	135 ± 19	145 ± 19	144 ± 17
**SUG4**	143 ± 26	159 ± 31	160 ± 32	156 ± 33	149 ± 31	151 ± 32	148 ± 30	149 ± 29	145 ± 28	144 ± 20
**SUG8**	143 ± 27	161 ± 33	166 ± 27	164 ± 32	146 ± 31	142 ± 31	137 ± 30	145 ± 28	138 ± 33	145 ± 30
**Arterial diastolic pressure (mmHg)**										
**SUG2**	51 ± 08	61 ± 14	67 ± 05	60 ± 09	63 ± 02	61 ± 04	70 ± 23	62 ± 17	61 ± 14	55 ± 14
**SUG4**	69 ± 19	63 ± 24	64 ± 17	65 ± 08	60 ± 10	69 ± 18	63 ± 16	62 ± 12	58 ± 14	57 ± 10
**SUG8**	63 ± 11	53 ± 16	50 ± 10	57 ± 24	54 ± 15	53 ± 14	49 ± 12	52 ± 17	54 ± 04	51 ± 04
**Arterial mean pressure (mmHg)**										
**SUG2**	65 ± 11	76 ± 15	84 ± 04	74 ± 10	77 ± 05	73 ± 08	82 ± 16	79 ± 19	78 ± 16	69 ± 12
**SUG4**	80 ± 21	74 ± 24	75 ± 22	74 ± 15	72 ± 12	82 ± 18	76 ± 18	76 ± 14	72 ± 15	70 ± 13
**SUG8**	74 ± 12	65 ± 16	61 ± 11	69 ± 25	70 ± 15	69 ± 14	62 ± 10	67 ± 15	73 ± 14	68 ± 03
**Arterial systolic pressure (mmHg)**										
**SUG2**	99 ± 16	113 ± 22	117 ± 10	107 ± 18	99 ± 10	100 ± 20	103 ± 15	108 ± 18	108 ± 18	94 ± 11
**SUG4**	104 ± 22	104 ± 20	106 ± 22	105 ± 11	99 ± 18	113 ± 23	101 ± 24	98 ± 18	98 ± 18	94 ± 18
**SUG8**	100 ± 15	90 ± 16	85 ± 14	99 ± 28	100 ± 13	100 ± 14	90 ± 11	95 ± 15	101 ± 08	100 ± 04
**End-tidal carbon dioxide (mmHg)**										
**SUG2**	36 ± 6	38 ± 5	38 ± 4	37 ± 5	36 ± 5	35 ± 6	35 ± 5	37 ± 6	36 ± 6	38 ± 4
**SUG4**	36 ± 6	34 ± 8	34 ± 4	34 ± 4	33 ± 4	32 ± 4	32 ± 4	33 ± 3	34 ± 4	34 ± 5
**SUG8**	37 ± 5	40 ± 6	41 ± 5	37 ± 6	37 ± 7	35 ± 6	36 ± 7	35 ± 6	35 ± 5	36 ± 5
**End-tidal sevoflurane concentration (%)**										
**SUG2**	2.6 ± 0.5	2.7 ± 0.5	2.7 ± 0.5	2.8 ± 0.4	2.7 ± 0.4	2.7 ± 0.4	2.8 ± 0.5	2.8 ± 0.4	2.9 ± 0.5	2.9 ± 0.2
**SUG4**	2.6 ± 0.3	2.6 ± 0.4	2.7 ± 0.3	2.7 ± 0.4	2.8 ± 0.4	2.7 ± 0.4	2.8 ± 0.5	2.8 ± 0.5	2.7 ± 0.4	2.6 ± 0.3
**SUG8**	2.8 ± 0.5	2.6 ± 0.5	2.6 ± 0.5	2.6 ± 0.5	2.7 ± 0.5	2.7 ± 0.5	2.7 ± 0.6	2.7 ± 0.5	2.6 ± 0.5	2.7 ± 0.5
**Temperature (°C)**										
**SUG2**	37.7 ± 1.1	37.9 ± 0.9	37.9 ± 0.9	37.6 ± 1.0	37.7 ± 1.1	37.6 ± 1.1	37.7 ± 1.1	37.6 ± 1.1	37.8 ± 1.1	37.8 ± 1.1
**SUG4**	37.7 ± 1.0	37.7 ± 0.9	37.6 ± 0.9	37.6 ± 0.9	37.6 ± 0.9	37.5 ± 0.8	37.6 ± 0.8	37.5 ± 0.8	37.6 ± 0.8	37.6 ± 0.7
**SUG8**	38.1 ± 0.3	38.1 ± 0.4	38.2 ± 0.4	38.1 ± 0.4	38.1 ± 0.5	38.1 ± 0.5	38.1 ± 0.4	38.0 ± 0.6	38.0 ± 0.6	38.1 ± 0.5

Legend: SUG2, 2 mg kg^−1^; SUG4, 4 mg kg^−1^; SUG8, 8 mg kg^−1^ of sugammadex; ROC, time after rocuronium administration (1–5 min); SUG, time after sugammadex administration (1–30 min).

## Data Availability

The original contributions presented in this study are included in the article. Further inquiries can be directed to the corresponding author.

## Supplementary Materials

The following supporting information can be downloaded at https://www.mdpi.com/article/10.3390/vetsci12121135/s1. Video S1: Electrode placement at the ulnar nerve.
